# Greenness exposure across the cancer continuum: The role of coronary heart disease

**DOI:** 10.1097/EE9.0000000000000520

**Published:** 2026-08-07

**Authors:** Saar Ashri, Gali Cohen, Lital Keinan-Boker, Osnat Itzhaki Ben Zadok, Itamar Shafran, David M. Broday, David M. Steinberg, Tamir Bental, Lihi Golan, Mika Moran, Ran Kornowski, Yariv Gerber

**Affiliations:** a Department of Epidemiology and Preventive Medicine, School of Public Health, Gray Faculty of Medical and Health Sciences, Tel Aviv University, Tel Aviv, Israel; b School of Public Health, University of Haifa, Haifa, Israel; c Israel Center for Disease Control, Israel Ministry of Health, Ramat Gan, Israel; d Department of Cardiology, Rabin Medical Center (Beilinson and Hasharon Hospitals), Petach Tikva, Israel; e Technion Center of Excellence in Exposure Science and Environmental Health, Technion Israel Institute of Technology, Haifa, Israel; f Department of Statistics and Operations Research, Tel Aviv University, Tel Aviv, Israel.

**Keywords:** Cardio-oncology, Cohort studies, Green space, NDVI

## Abstract

**Background:**

Whether residential greenness affects cancer outcomes across the cancer continuum and whether comorbid coronary heart disease (CHD) confers differential vulnerability remains an open question. We evaluated greenness-cancer associations and CHD effect modification in a pooled multicohort study.

**Methods:**

Four cohorts were pooled: two CHD patient registries and two Israeli National Health and Nutrition Surveys. Cancer and mortality data were obtained from national registries. Greenness was estimated using the Normalized Difference Vegetation Index (NDVI) within a 300 m buffer. Incident cancer cases were 1:1 matched to cancer-free controls by age, sex, cohort, and enrollment year and followed for second primary cancer incidence and mortality. Conditional logistic and Cox regression models estimated hazard ratios (HRs) per 1- standard deviation NDVI increase; CHD effect modification was tested.

**Results:**

Overall, 17,047 individuals were pooled, including 1,661 prevalent cancer cases. Over a median of 13.6 years, 2,481 incident cancer cases [age 74 (11.3) years, 27% females] were identified; 1,387 died. Prevalent cases [age 75 (10.9) years, 33% females] had 1,125 deaths over a median of 15.0 years. Greenness associations were specific to postcancer survival: NDVI-300 was not associated with first or second primary cancer incidence [HRs (95% confidence intervals) = 0.96 (0.90, 1.02) and 1.04 (0.93, 1.16), respectively]. NDVI-300 was inversely associated with postcancer mortality [HRs (95% confidence intervals) = 0.93 (0.88, 0.99) and 0.92 (0.86, 0.98) in incident and prevalent groups]. The association was consistent regardless of CHD status (all *P*-for-effect-modification > 0.05).

**Conclusions:**

While no association was observed for cancer incidence, residential greenness was consistently associated with lower postcancer mortality, regardless of CHD status.

**What this study adds**

We studied over 17,000 individuals from diverse backgrounds, examining whether residential greenness—measured via satellite imagery—is associated with cancer incidence, second primary cancer, and postdiagnosis survival. Each standard deviation increase in greenness was associated with approximately an 8% lower mortality risk after cancer diagnosis, both in incident and prevalent cancer patients. No association was found with the incidence of cancer or a second primary malignancy. The association was not modified by preexisting coronary heart disease. Spanning multiple cancer outcomes across the cancer continuum in a diverse population, these findings offer novel evidence linking greener residential environments to better cancer survival.

## Introduction

Recent advances in cancer detection and treatment have resulted in a rapidly growing survivor population.^1,2^ This demographic shift has reframed cancer as a chronic condition, spanning incidence, treatment sequelae, metachronous malignancies, and long-term survivorship—the cancer continuum.^3,4^ Cancer and coronary heart disease (CHD) share risk factors and convergent pathophysiology, including systemic inflammation, oxidative stress, and cardiometabolic dysregulation.^5,6^ Cancer-CHD co-occurrence reflects aging populations with compounded vulnerability, further amplified by cardiotoxic cancer therapies.^7–9^ The relationship is multifaceted: preexisting CHD is associated with cancer at metastatic stages and adversely affects treatment candidacy and postdiagnosis survival.^10–14^ Conversely, cancer survivors face an elevated risk of incident CHD through shared biological pathways and therapy-related cardiotoxicity.^15^ Despite this, the influence of environmental factors on cancer outcomes in individuals with concurrent cardiovascular disease remains largely uncharacterized.

Residential greenness, commonly quantified via the satellite-derived Normalized Difference Vegetation Index (NDVI), has emerged as a prominent environmental exposure associated with reduced mortality, rehabilitation, and social cohesion.^16^ Large cohort studies consistently link higher greenness to reduced all-cause and cardiovascular mortality,^17–19^ and large-scale analyses have extended these associations to cause-specific endpoints, including lung and breast cancer mortality.^20,21^ Notably, cancer mortality is not interchangeable with incidence; it reflects a composite of incidence, stage, treatment, and postdiagnosis survival. Evidence on cancer incidence has been more mixed, with site-specific but inconsistent overall incidence associations across cohorts.^22–24^ Evidence on cancer survivorship is particularly sparse; in our prior cohort study, residential NDVI was independently associated with lower all-cause mortality among cancer survivors with established CHD.^25^

Yet critical gaps remain. Studies have examined cancer incidence or postdiagnosis mortality in isolation; second primary cancer incidence remains uninvestigated. No study has evaluated greenness across the full cancer continuum in a single framework, nor the role of cardiovascular vulnerability, despite its well-documented prognostic relevance in cancer survivorship.^7^ Using a pooled multicohort design of Israeli adults linked to national cancer and mortality registries, this study examined greenness in relation to cancer and second primary cancer incidence, postdiagnosis mortality, and whether baseline CHD modifies these associations.

## Methods

### Study design and setting

**Figure 1.**
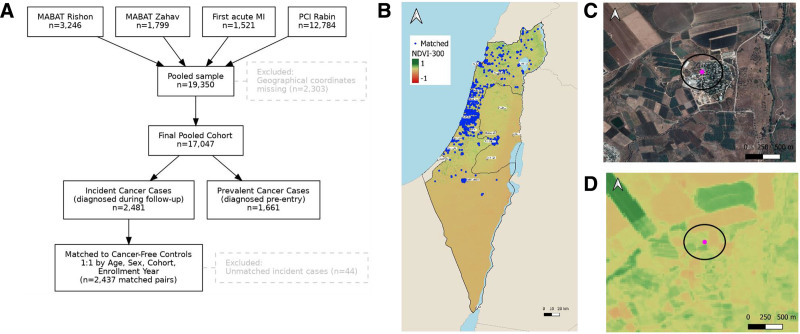
Study population, design, and assessment of exposure to residential greenness. A, Cohort assembly and study design. B, Residential distribution of individuals included in the matched cohort (blue dots) and mean annual normalized difference vegetation index at a 300 m buffer (NDVI-300) measured using NASA’s Landsat satellite series, during 2007 as an exemplary year. C, A satellite image of a chosen participant’s location. D, NDVI measurements within a 300-m buffer around their residence.

This is a retrospective cohort study utilizing a pooled dataset of four samples,^26^ including two CHD patient registries and two nationwide population samples (n = 19,350; Figure 1). The largest cohort comprised all consecutive patients (n = 12,784) undergoing percutaneous coronary intervention (PCI) at Rabin Medical Center, a tertiary center in central Israel, between 2004 and 2014. The second CHD cohort included all individuals aged <65 years admitted with myocardial infarction (MI) to one of eight hospitals across central Israel, between 1992 and 1993 (n = 1,521). Two Israeli population samples were also included: “Mabat Rishon,” comprising 3,246 individuals aged 25–64 (inception years: 1999–2001), and “Mabat Zahav,” comprising 1,799 individuals aged 65+ (inception years: 2005–2006). Individuals with missing information on either greenness exposure, cancer, or mortality were excluded from analyses (Figure 1).

The cohort was divided into prevalent cancer cases (diagnosed before the index date) and incident cancer cases (diagnosed during follow-up). To examine the instantaneous risk of cancer incidence, a nested case-control design was employed, matching incident cases to an equal number (1:1) of cancer-free controls using risk set sampling based on age, sex, cohort, and enrollment year. Controls were sampled from all individuals in the risk set who were cancer-free at the time of each case’s diagnosis; subsequent cancer development did not disqualify a control, and individuals could contribute to multiple risk sets. Both groups were followed from the index date for postdiagnosis mortality. Second primary cancer incidence was additionally examined. Combined, the analysis spans multiple cancer outcomes across the cancer continuum. Across all analyses, baseline CHD status was the primary effect modifier. All aspects of this study were approved by the relevant ethics committees; patients’ consent was waived (approval #SMC-5423-18).

### Assessment of greenness exposure

Residential greenness at each participant’s home address was estimated using NDVI, a satellite-based index, as previously described.^25,27^ In summary, NDVI measures vegetation density by calculating the ratio between the difference and sum of near-infrared (0.7–1.1 μm) and red light (0.4–0.7 μm) reflectance, approximating photosynthesis. NDVI values range from −1.0 to 1.0, with higher values indicating denser vegetation.^28^ NDVI was derived from Landsat 5 L2 C2 (1992–2012) and Landsat 8 L2 C2 (2013–2019) satellite imagery (30m spatial resolution), downloaded from the United States Geological Survey EarthExplorer platform. Annual values represent the mean of cloud-free images (<10% cloud cover) acquired between March and May of each year, corresponding to the Israeli spring phenological season when vegetation contrast is maximal.^29^ Measurements were averaged across all available years from each cohort’s inception through 2019 within a 300-m buffer radius (Figure 1), representing the directly accessible environment.^18,30,31^ Annual measurements were averaged into a single cumulative metric, with missing yearly observations excluded from individual averages; temporal reliability of the multiyear average was assessed using intraclass correlation coefficients,^32^ demonstrating high interyear consistency (intraclass correlation coefficient = 0.94–0.95).

### Cancer and mortality ascertainment

Information on cancer diagnoses was retrieved from the Israeli National Cancer Registry (INCR), covering Israel’s entire population since 1982, with mandatory reporting for all malignant tumors as well as in situ tumors, borderline tumors, and central nervous system benign tumors (estimated 97% completeness for solid tumors, 87% for hematopoietic malignancies).^33^ Data included diagnosis dates and International Classification of Diseases, 10th edition (ICD-10) codes (proxy for ICD-O-3). Cancer diagnoses were further grouped into organ-system subtypes (e.g., digestive, respiratory, and urinary) following the anatomical structure of ICD-10 Chapter II (Supplemental Table 1; https://links.lww.com/EE/A457). The index date was set at cohort enrollment (MI/PCI date, or interview date for population samples). For incident cases, the index date was subsequently updated to the date of cancer diagnosis. All individuals were followed for second primary cancer incidence and all-cause and cause-specific mortality through December 31, 2021. Data linkage was facilitated through TIMNA, Israel’s Ministry of Health research platform. Causes of death were available for 6,464 individuals (92% of mortality cases) and were adjudicated using ICD-10 codes; cardiopulmonary mortality includes causes of death from chapters I & J and cancer-related mortality from chapters C & D.^19^

### Baseline covariates and coronary heart disease ascertainment

Baseline covariates included age, sex, and ethnicity (Jewish/Arab). Residential socioeconomic status (rSES) was determined by the Central Bureau of Statistics based on a 2008 census, with scores ranging between 1 and 20; lower values represented deprived residential areas. Smoking status was self-reported (current smoking or no smoking). Diabetes mellitus, hypertension, and prior stroke were ascertained by treating physicians (registries) or self-reported (population samples). Long-term residential NOx levels, a proxy for traffic-related air pollution, were estimated using a high-resolution land use regression model, previously described.^34^ We defined CHD as clinically manifest coronary disease, including acute coronary events and coronary revascularization. All registry participants were assigned baseline CHD; population-sample ascertainment was self-reported at baseline.

### Statistical analysis

Baseline characteristics by NDVI quartiles are presented as percentages for categorical variables and means (standard deviation [SD]) for continuous variables. Spatial balance of matching was verified using a logistic generalized additive model with bivariate spatial smoothing.^35^ Median follow-up was estimated using the reverse Kaplan–Meier method.^36^ Conditional logistic regression estimated hazard ratios (HRs) and 95% confidence intervals (CIs) for cancer incidence per 1-SD NDVI increase. Second primary cancer and all-cause mortality were modeled using Cox regression with age as the timescale and cohort strata to account for differences in baseline hazards. For incident cases, follow-up began at the age of cancer diagnosis; for prevalent cases, a counting process formulation with age at cohort entry as the delayed-entry point was applied to account for left truncation.^37^ For second primary cancer, incident and prevalent cases were pooled with cancer-group strata. Cause-specific Cox regression models were constructed for cardiopulmonary and cancer-related mortality, with mortality from other causes treated as a competing risk; heterogeneity across causes was tested using Lunn–McNeil’s approach.^38^ The proportional hazards assumption was tested using the Schoenfeld residuals, with no violations found. An unadjusted analysis was initially conducted, followed by multivariable adjustment for sociodemographic, clinical, and behavioral risk factors (Arab ethnicity, rSES, smoking, diabetes mellitus, hypertension, prior stroke, and residential NO_x_ levels). To assess organ-system heterogeneity, site-specific estimates and Cochran’s Q were derived for cancer incidence and all-cause mortality (incident and prevalent groups); second primary cancers were excluded due to insufficient subtype-specific counts. CHD vulnerability was assessed via stratified analyses and NDVI × CHD interaction terms in fully adjusted models, per established recommendations.^39^ For cancer incidence models, effect modification was tested in fully adjusted models with group-specific NDVI parameterization. Sensitivity analyses included repeating primary models with 1:2 matching, deriving cohort-stratified estimates with formal heterogeneity testing, repeating primary models with different NDVI buffers, modeling NDVI nonlinearly using penalized splines, excluding individuals with self-reported CHD (due to potential self-report misclassification), and excluding participants with follow-up shorter than 2 years.^40^ We also tested the interaction between NDVI and CHD severity (number of diseased vessels in PCI; Killip class in MI) for postdiagnosis mortality within each registry cohort separately. Analysis was performed using R Statistical Software (v4.3.0; R Core Team, R Foundation for Statistical Computing, Vienna, Austria).

## Results

### Study population

The final pooled sample included 17,047 participants with complete exposure information across the four cohorts [mean (SD) age 64.11 (15.35) years; 5,287 (31.0%) females]. During a median follow-up of 13.6 (95% CI: 13.4, 13.7) years, 1,661 (9.7%) were classified as prevalent cancer cases at enrollment, 2,481 (14.6%) were identified as incident cases, and 12,905 (75.7%) remained cancer-free. The mean (SD) annual NDVI-300 exposure across the pooled population was 0.12 (0.03). Baseline clinical and behavioral characteristics of the pooled cohort, stratified by cancer status, are provided in Supplemental Table 2; https://links.lww.com/EE/A457. The nested case-control analysis included 4,874 participants, consisting of 2,437 incident cancer cases matched to an equal number of controls who were cancer-free at the time of matching [mean (SD) age 73.9 (11.3) years; 1,290 (26.5%) females; 44 cases could not be matched]. Incident cancer cases were more likely to be current smokers, to have higher rSES, and were less likely to be of Arab ethnicity compared with controls (Table 1). NDVI-300 was comparable between incident cases and matched controls (0.12 for both; *P* = 0.11). The spatial distribution of sampled controls did not differ meaningfully from nonsampled individuals in the pooled cohort (*P* = 0.21).

**Table 1.** Baseline characteristics of cancer cases and matched control.

Baseline Characteristic	Cancer Cases (N = 2,437)	Matched Controls (N = 2,437)	Overall (N = 4,874)
NDVI^a^	0.118 (0.030)	0.119 (0.030)	0.118 (0.030)
Age, yr	73.9 (11.3)	73.9 (11.4)	73.9 (11.4)
Female sex	645 (26.5)	645 (26.5)	1,290 (26.5)
rSES^b^	11.0 (4.7)	10.5 (4.8)	10.7 (4.7)
Current smoking	867 (35.6)	736 (30.3)	1,603 (32.9)
Prevalence of Diabetes mellitus	841 (34.5)	797 (32.8)	1,638 (33.6)
Prevalence of Hypertension	1,470 (60.6)	1,443 (59.7)	2,913 (60.1)
Prior stroke	98 (4.0)	103 (4.2)	201 (4.1)
Baseline CHD	1,884 (77.3)	1,889 (77.5)	3,773 (77.4)
NO_x_^c^_,_ ppb	22.2 (10.8)	21.6 (10.5)	21.9 (10.7)

Data are mean (SD) or count (%).

ppb, parts per billion.

a Normalized Difference Vegetation Index, a satellite-derived unitless measure of vegetative density ranging from −1 to 1, estimated within a 300 m residential buffer using Landsat imagery (30 m resolution); higher values indicate greater greenness.

b Residential socioeconomic status, derived from the Israeli Central Bureau of Statistics unitless socioeconomic cluster index, scored on a scale of 1–20; higher values indicate higher socioeconomic status.

c Nitrogen oxides concentration (parts per billion), estimated at the residential address using a land-use regression model as a proxy for traffic-related air pollution.

### Residential greenness and incidence of cancer and second primary cancer

In the conditional logistic regression models, residential greenness was not significantly associated with all-site cancer incidence. The crude HR (95% CI) was 0.95 (0.90, 1.01; Table 2) per 1-SD increase in NDVI-300, which remained stable following adjustment for potential confounders (HR = 0.96; 95% CI: 0.90, 1.02). These findings were consistent across all examined buffers (Supplemental Table 3; https://links.lww.com/EE/A457). Baseline CHD status did not modify this association (*P*-for-effect modification = 0.58). Additionally, no heterogeneity by cancer site was identified (*P* = 0.22; Supplemental Figure 1; https://links.lww.com/EE/A457).

**Table 2.** Hazard ratios (95% confidence intervals) for cancer incidence, second primary incidence, and postcancer diagnosis mortality associated with a 1-SD increase in normalized difference vegetation index in a 300 m buffer (NDVI-300).

Group	N	Cases	Unadjusted	Adjusted	*P*-for-Effect Modification
Cancer incidence					
Overall	4,874	2,437	0.95 (0.90, 1.01)	0.96 (0.90, 1.02)	0.58
No CHD	1,101	553	1.02 (0.89, 1.16)	0.96 (0.82, 1.12)	
CHD	3,773	1,884	0.94 (0.88, 1.00)	0.96 (0.89, 1.03)	
Second primary cancer incidence					
Overall	4,098	352	1.01 (0.91, 1.12)	1.04 (0.93, 1.16)	0.56
No CHD	752	72	0.93 (0.74, 1.19)	0.97 (0.73, 1.28)	
CHD	3,346	280	1.03 (0.92, 1.16)	1.04 (0.92, 1.17)	
Mortality among incident cases					
Overall	2,437	1,387	0.93 (0.88, 0.99)	0.93 (0.88, 0.99)	0.20
No CHD	553	269	0.86 (0.75, 0.99)	0.95 (0.82, 1.11)	
CHD	1,884	1,118	0.95 (0.89, 1.01)	0.94 (0.88, 1.00)	
Mortality among prevalent cases					
Overall	1,661	1,125	0.92 (0.86, 0.97)	0.92 (0.86, 0.98)	0.13
No CHD	199	119	0.75 (0.60, 0.94)	0.69 (0.55, 0.88)	
CHD	1,462	1,006	0.93 (0.88, 1.00)	0.93 (0.88, 1.00)	

Adjusted for sex, residential socioeconomic status, smoking, chronic exposure to air pollution (NO_x_), diabetes, hypertension, and prior stroke.

We further examined second primary cancer incidence and identified 352 (8.6%) events. NDVI-300 was not associated with second primary incidence (crude HR = 1.01; 95% CI: 0.91, 1.12; adjusted HR = 1.04; 95% CI: 0.93, 1.16). The greenness-second primary association was similarly not modified by baseline CHD status (*P*-for-interaction = 0.56).

### Mortality after cancer diagnosis

We identified 1,387 (56.9%) deaths among incident cancer cases during a median follow-up period of 9.3 (95% CI: 8.9, 9.8) years and 1,125 (67.7%) deaths among prevalent cases during a median follow-up of 15.0 (95% CI: 14.6, 15.2) years. NDVI-300 was inversely associated with postdiagnosis all-cause mortality in the incident (HR per 1-SD increase in NDVI-300 = 0.93, 95% CI: 0.88, 0.99) and prevalent (HR = 0.92, 95% CI: 0.86, 0.97) groups. The associations did not attenuate following adjustment [HRs (95% CIs) 0.93 (0.88, 0.99) and 0.92 (0.86, 0.98), respectively]. Baseline CHD status did not modify the greenness–mortality association in either the incident or prevalent groups (*P*-for-interaction = 0.20 and 0.13, respectively). Heterogeneity by cancer site was not evident in both groups (*P* = 0.96 and 0.53, respectively; Supplemental Figure 1; https://links.lww.com/EE/A457).

We additionally examined cause-specific mortality; point estimates suggested inverse associations for cardiopulmonary and cancer-related mortality in both incident and prevalent groups, though associations attenuated following adjustment and did not differ significantly by cause of death in either group (*P*-for-heterogeneity = 0.85 and 0.50, respectively; Supplemental Table 4; https://links.lww.com/EE/A457).

### Sensitivity analysis

**Figure 2.**
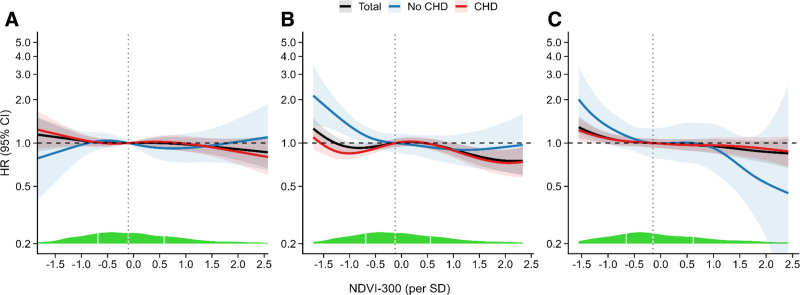
Association between residential greenness and cancer outcomes. Penalized spline-based unadjusted hazard ratios (HRs) and 95% confidence intervals (95% CIs; shaded area) for (A) cancer incidence, (B) mortality in the incident group, and (C) mortality in the prevalent group associated with a 1-SD change in mean annual normalized difference vegetation index at a 300 m buffer (NDVI-300). The histogram at the bottom shows the relative overall distribution of NDVI; the dotted vertical line denotes the median. The *y* axis is presented on a logarithmic scale.

Repeating cancer incidence models with 1:2 matching yielded results consistent with the primary analysis, as did cohort-stratified analyses across outcomes (all *P*-for-heterogeneity > 0.05; Supplemental Figure 2; https://links.lww.com/EE/A457). We tested the linearity assumption for the association between NDVI-300 and cancer incidence and mortality in both incident and prevalent groups using penalized spline terms, without significant nonlinearity detected (*P*-for-nonlinearity = 0.62, 0.10, and 0.33, respectively; Figure 2). Additionally, we restricted analysis to individuals with at least 2 years of potential follow-up, with no apparent attenuation observed (Supplemental Table 5; https://links.lww.com/EE/A457). We further excluded 732 individuals with self-reported CHD (19.3% of all CHD individuals) from analysis, similarly with no attenuation observed (Supplemental Table 6; https://links.lww.com/EE/A457). Exploratory NDVI × CHD severity interactions within registry cohorts were largely consistent (Supplemental Figure 3; https://links.lww.com/EE/A457).

## Discussion

This study examined residential greenness across the cancer continuum, from incidence and second primary cancer incidence to postdiagnosis mortality. While greenness was not associated with the risk of cancer or second primary cancer incidence, it was associated with a lower risk of all-cause mortality after a cancer diagnosis. This beneficial association was consistently observed in both incident and prevalent cancer cases and was not modified by baseline CHD status. These findings suggest that the observed association of residential greenness may be more pronounced for postdiagnosis mortality than for the incidence of initial or second primary cancers, indicating a potentially greater relevance at later stages of the cancer continuum.

The mechanisms linking residential greenness to cancer outcomes are likely multifactorial. Greenness may reduce cancer initiation risk through hazard mitigation, particularly traffic-related air pollution (e.g., particulate matter 2.5 and NOx), a known carcinogenesis driver.^41,42^ On the other hand, cancer initiation is mechanistically heterogeneous^43^ and characterized by long latency,^44^ such that greenness-related effects may be susceptible to exposure timing.^45^ As NDVI reflects a stable, multiyear average of residential greenness, our estimates likely reflect chronic exposure that preceded the start of follow-up. Postdiagnosis benefits likely reflect the rehabilitative pathways described by Markevych et al^16^—including capacity restoration (physical activity and psychological well-being) and social cohesion—which are particularly salient during cancer and CHD survivorship, when deconditioning,^46^ cardiometabolic burden,^47,48^ and psychological distress^49^ are most pronounced. Residential greenness may additionally serve as a marker for a broader set of neighborhood characteristics, including walkability, environmental quality, and social conditions, that are difficult to fully disentangle in observational settings.

The absence of an apparent association with initial or second primary cancer incidence suggests that, in this setting, greenness may play a more limited role in carcinogenesis than in survival. Evidence from cohort studies demonstrates site specificity, with associations diluted when aggregated at an all-site endpoint; for example, Huang et al^22^ found no association with overall cancer incidence in ~400,000 Taiwanese adults, yet observed lower risks of lung and prostate cancer specifically. In a 21-year Israeli cohort, Kayyal-Tarabeia et al^24^ reported inverse associations between NDVI and lung, bladder, and genital cancer incidence; an association with all-site cancer was not reported. Unlike these studies, exploratory site-specific secondary analyses in the present study yielded no statistically significant heterogeneity across organ systems, suggesting a potentially limited role of greenness in carcinogenesis in this population rather than masking by dilution of site-specific effects at the aggregated endpoint.

Second primary cancer represents a distinct and underexplored domain on the cancer continuum, and to our knowledge, this is among the first epidemiologic studies to examine its relationship with residential greenness.^50^ Beyond residual tumor biology, second malignancies are shaped by elevated carcinogenic stress related to the treatment of the primary tumor,^51,52^ tumor dormancy reactivation^53^ as well as sustained psychological burden.^54^ Although greenness, through stress attenuation and autonomic restoration, could theoretically modulate this risk, we did not observe such an association. This may reflect outcome heterogeneity across tumor sites and limited statistical power in our cohort.

Greenness was associated with lower case fatality in both incident and prevalent cancer groups. While results in the prevalent group are consistent with the literature,^25^ they represent a treatment-experienced population prone to the healthy survivor effect; the association in newly diagnosed cases therefore extends this evidence beyond survivor selection. Coleman et al^19^ similarly reported an inverse greenness–postcancer diagnosis mortality association in a large US cohort, with associations specific to cancer-related rather than cardiopulmonary mortality. In contrast, cause-specific analyses in the current study did not yield heterogeneous associations across these endpoints, suggesting no differential pathway predominance, though power to detect cause-specific differences was limited.

CHD status did not modify the observed greenness associations for any of the three outcomes examined: cancer incidence, second primary cancer incidence, and postdiagnosis mortality. For cancer incidence and second primary cancer, the absence of both a main greenness association and CHD interaction is consistent with no meaningful role for cardiometabolic burden in differentially shaping greenness-related carcinogenic pathways in this population. For postdiagnosis mortality, where a clear inverse association was observed, the null interaction carries more direct interpretive weight. Cancer and CHD share physiological vulnerabilities that greenness may attenuate, suggesting that cancer patients with established CHD might derive amplified benefit given their heightened cardiometabolic burden. Conversely, their higher competing mortality risk could obscure a differential effect, the “reverse epidemiology” paradox.^55^ Neither pattern emerged; the consistent association across CHD strata instead suggests that the pathways linking greenness to postdiagnosis survival operate broadly across the cardio-oncology population, rather than being contingent on cardiovascular comorbidity. The pooled design of this study places structural constraints on this testing; however, registry cohorts contribute to the CHD+ estimate but cannot generate CHD-discordant pairs, so cross-CHD contrasts rely on between-cohort variation.

This study has several notable strengths. First, we utilized a large, pooled sample of individuals from four diverse cohorts, including both national health surveys and patient registries, which allowed for a broad representation of participants with and without baseline cardiovascular disease. Second, the longitudinal design featured an exceptionally long follow-up period, with a median of over 15 years, enhancing power to assess long-term mortality and incident cancer events. Third, the use of high-quality national registry data from the INCR and the Ministry of Health ensured objective and accurate ascertainment of outcomes. Furthermore, the high-resolution (30 m) satellite-derived NDVI data offered an objective measurement of residential greenness exposure at the residential level, a well-accepted methodology.^28^ While NDVI is commonly assessed cross-sectionally or over short intervals in existing literature,^31^ we averaged long-term annual estimated NDVI to characterize chronic residential greenness exposure.

Several limitations warrant consideration. First, we pooled heterogeneous cohorts, possibly introducing an aggregation bias;^56^ however, cohort-stratified analysis did not reveal heterogeneity. Greenness exposure was assessed at the residential address, without capturing daily mobility or residential relocation.^16,25^ On the other hand, the older age of participants likely reduces exposure misclassification.^57^ Individual-level data on exposures such as body mass index, physical activity, diet, and social well-being were unavailable; these may represent mediators rather than pure confounders.^58^ A recent study suggests substantial differences in physical activity levels by CHD status,^59^ with coronary patients often engaging in lower levels of activity, potentially resulting in reduced exposure to outdoor greenness. Similarly, CHD severity could not be harmonized across cohorts. While we adjusted for residential SES, individual-level social determinants of health were unavailable. Regarding healthcare access specifically, all participants benefit from national universal healthcare coverage, making differential access an unlikely explanation for the observed associations; however, health literacy and individual-level SES remain unaccounted for. The absence of data on cancer stage or metastatic status precluded analysis of greenness in relation to disease progression; true cancer recurrence data were also unavailable. This study may be subject to left censoring, and NDVI does not reflect green space quality characteristics. Other cardiovascular conditions beyond CHD were not accounted for and remain to be examined. Reverse causality is unlikely, given consistent results in sensitivity analyses excluding short follow-up times.

## Conclusions

In this pooled sample of individuals with and without CHD, residential greenness was associated with a lower risk of mortality following a cancer diagnosis, but not with initial cancer or second primary cancer incidence—a pattern consistent with associations concentrated at the survivorship end of the cancer continuum. In an exploratory analysis, baseline CHD status did not appear to modify the greenness–mortality association, suggesting that residential greenness may confer protective associations across the cardio-oncologic continuum. These findings underscore the potential of residential greenness as a modifiable factor associated with cancer survivorship.

## Conflicts of interest statement

The authors declare that they have no conflicts of interest with regard to the content of this report.

## Supplementary Material
