# Supplementary material for: Greenness exposure across the cancer continuum: The role of coronary heart disease
Source: Environ Epidemiol. 2026 Aug 7;10(4):e520. doi: 10.1097/EE9.0000000000000520 (PMC13456911; doi:10.1097/EE9.0000000000000520)
Supplement: Supplementary file 1 [file ee9-10-e520-s001.pdf]

Greenness Exposure Across the Cancer Continuum: The Role of Coronary Heart Disease

SUPPLEMENTAL APPENDIX

Supplemental Tables

Supplemental Table 1. Cancer ascertainment into organ systems. Page 2

Supplemental Table 2. Baseline characteristics across greenness exposure groups in the total cohort. Page 4

Supplemental Table 3. Hazard ratios (95% CIs) for cancer outcomes associated with normalized difference vegetation index (NDVI) in 100-, 300- & 800-m buffers. Page 5

Supplemental Table 4. Hazard ratios (95% CIs) for cause-specific mortality associated with 1-SD increase in normalized difference vegetation index in 300m buffer (NDVI-300). Page 6

Supplemental Table 5. Hazard ratios (95% CIs) for cancer outcomes associated with NDVI in 300-m buffer, excluding participants with follow-up shorter than two years. Page 7

Supplemental Table 6. Hazard ratios (95% CIs) for cancer outcomes associated with 1-SD change in normalized difference vegetation index (NDVI) in 300-m buffers, excluding participants with self-reported coronary heart disease (CHD). Page 8

Supplemental Figures

Supplemental Figure 1. Heterogeneity analysis for the association between NDVI and cancer incidence and post-cancer mortality, stratified by organ system. Page 9

Supplemental Figure 2. Cohort-stratified associations between 1-SD change in NDVI and cancer incidence with 1:1 and 1:2 matching and post-cancer mortality among the prevalent group and the incident group. Page 10

Supplemental Figure 3. Associations between 1-SD change in NDVI and post-cancer mortality by CHD severity stratum within registry cohorts. Page 11

Other material

List of R packages used for the analysis. Page 12

Supplemental Table 1. Cancer ascertainment into organ systems.

#	System	Organs	ICD-10-CM codes	Incident cases	Prevalent cases
1	Lip, Oral Cavity and Pharynx	Lip, Base of tongue, Other and unspecified parts of tongue, Gum, Floor of mouth, Palate, Other and unspecified parts of mouth, Parotid gland, Other and unspecified major salivary glands, Tonsil, Oropharynx, Nasopharynx, Pyriform sinus, Hypopharynx, Other and ill-defined sites in the lip, oral cavity and pharynx	C00, C01, C02, C03, C04, C05, C06, C07, C08, C09, C10, C11, C12, C13, C14	37	60
2	Digestive Organs	Esophagus, Stomach, Small intestine, Colon, Rectosigmoid junction, Rectum, Anus and anal canal, Liver, Gallbladder, Other and unspecified parts of biliary tract, Pancreas, Other ill-defined digestive organs	C15, C16, C17, C18, C19, C20, C21, C22, C23, C24, C25, C26	529	298
3	Respiratory System and Intrathoracic Organs	Nasal cavity and middle ear, Accessory sinuses, Larynx, Trachea, Bronchus and lung, Thymus, Heart, mediastinum and pleura, Other and ill-defined sites in the respiratory and intrathoracic organs	C30, C31, C32, C33, C34, C37, C38, C39	309	89
4	Bones, Joints and Articular Cartilage	Bone and articular cartilage of limbs, Other and ill-defined sites in the respiratory and intrathoracic organs	C40, C41	3	9
5	Skin	Malignant melanoma of skin, Other and unspecified malignant neoplasm of skin	C43, C44	155	129
6	Mesothelial and Soft Tissue	Mesothelial and Soft Tissue	C45	0	0
7	Kaposi Sarcoma	Kaposi sarcoma	C46	0	0
8	Peripheral Nerves and Autonomic	Peripheral nerves and autonomous nervous system, Retroperitoneum	C47, C48, C49	16	15

	nervous system, Retroperitoneum, Peritoneum, and Soft Tissues	and peritoneum, Other connective and soft tissue			
9	Breast and Female Genital Organs	Breast, Vulva, Vagina, Cervix uteri, Corpus uteri, Uterus, part unspecified, Ovary, Other and unspecified female genital organs, Placenta	C50, C51, C52, C53, C54, C55, C56, C57, C58	265	273
10	Male Genital Organs	Penis, Prostate, Testis, Other and unspecified male genital organs	C60, C61, C62, C63	294	273
11	Urinary Tract	Kidney, Renal pelvis, Ureter, Bladder, Other and unspecified urinary organs	C64, C65, C66, C67, C68	278	242
12	Eye, Brain and Other Parts of the Central Nervous System	Eye and adnexa, Meninges, Brain, Spinal cord, cranial nerves, and other parts of the central nervous system	C69, C70, C71, C72	50	47
13	Thyroid, Other Endocrine Glands, and Ill-defined Sites	Thyroid gland, Adrenal gland, Other endocrine glands and related structures, Other and ill-defined sites	C73, C74, C75, C76	37	62
14	Lymph nodes	Lymph nodes	C77	69	80
15	Unknown primary	Unknown primary	C80	77	19
	ICD-code not provided			397	71

Supplemental Table 2. Baseline characteristics across greenness exposure groups in the total cohort.

	Overall (N = 17,047)	Tertile 1, lowest (N = 5,626)	Tertile 2 (N = 5,795)	Tertile 3, highest (N = 5,626)	p
**NDVI-300**	0.12 (0.03)	0.09 (0.01)	0.11 (0.01)	0.15 (0.02)	< .01
**Age, years**	64.11 (15.4)	64.38 (15.6)	64.21 (15.0)	63.75 (15.4)	NS
**Female sex**	5,287 (31.0)	1,842 (32.8)	1,827 (31.5)	1,618 (28.8)	< .01
**Arab ethnicity**	1,934 (11.4)	921 (16.4)	709 (12.2)	304 (5.4)	< .01
**rSES**	10.45 (4.78)	9.89 (3.86)	10.90 (4.45)	10.54 (5.78)	< .01
**Smoking**	5,516 (32.4)	1,863 (33.2)	1,909 (33.0)	1,744 (31.1)	< .05
**DM**	5,905 (34.7)	1,971 (35.1)	2,021 (34.9)	1,913 (34.1)	NS
**Hypertension**	10,093 (59.7)	3,301 (59.1)	3,515 (61.1)	3,277 (58.7)	< .05
**Prior stroke**	854 (5.0)	324 (5.8)	289 (5.0)	241 (4.3)	< .01
**Baseline CHD**	3,790 (22.2)	1,297 (23.1)	1,232 (21.3)	1,261 (22.4)	< .05
**NO_x, ppb**	21.16 (10.5)	23.53 (11.5)	21.58 (10.2)	18.37 (8.8)	< .01

Data are mean (SD) or %. P-values denote significance of correlations with NDVI-300.

NDVI = normalized difference vegetation index; rSES = residential socioeconomic status; DM = diabetes mellitus; CHD = coronary heart disease; ppb = parts per billion.

Supplemental Table 3. Hazard ratios (95% CIs) for cancer and second primary cancer incidence, and post-cancer mortality associated with 1-SD increase in normalized difference vegetation index in 100m, 300m and 800m buffers.

	**Unadjusted**	**Adjusted**
**Cancer incidence**		
100m	0.95 (0.90, 1.01)	0.95 (0.89, 1.01)
300m	0.95 (0.90, 1.01)	0.96 (0.90, 1.02)
800m	0.97 (0.91, 1.02)	0.98 (0.92, 1.05)
**Second primary cancer incidence**		
100m	1.02 (0.92, 1.13)	1.05 (0.94, 1.17)
300m	1.01 (0.91, 1.12)	1.04 (0.93, 1.16)
800m	1.03 (0.92, 1.14)	1.06 (0.95, 1.18)
**Mortality among incident cases**		
100m	0.94 (0.89, 1.00)	0.93 (0.88, 0.99)
300m	0.93 (0.88, 0.99)	0.93 (0.88, 0.99)
800m	0.94 (0.89, 1.00)	0.92 (0.87, 0.98)
**Mortality among prevalent cases**		
100m	0.92 (0.87, 0.98)	0.93 (0.87, 0.99)
300m	0.92 (0.86, 0.97)	0.92 (0.86, 0.98)
800m	0.92 (0.87, 0.98)	0.92 (0.86, 0.98)

* Adjusted for sex, residential socioeconomic status (rSES), smoking, chronic exposure to air pollution (NO_x), diabetes mellitus, hypertension and prior stroke.

Supplemental Table 4. Hazard ratios (95% CIs) for cause-specific mortality associated with 1-SD increase in normalized difference vegetation index in 300m buffer (NDVI-300).

	Cardiopulmonary mortality			Cancer mortality		
	N / Cases	Unadjusted	Multivariable adjustment	N / Cases	Unadjusted	Multivariable adjustment
Incident group	2,437 / 179	0.95 (0.81, 1.11)	0.98 (0.83, 1.16)	2,437 / 828	0.93 (0.86, 1.00)	0.92 (0.85, 0.99)
p-for-heterogeneity = .85						
Prevalent group	1,661 / 312	0.90 (0.80, 1.01)	0.90 (0.80, 1.01)	1,661 / 373	0.94 (0.85, 1.05)	0.95 (0.86, 1.06)
p-for-heterogeneity = .50						

Models were adjusted for sex, residential socioeconomic status, smoking, chronic exposure to air pollution (NO_x), diabetes, hypertension and prior stroke.

Supplemental Table 5. Hazard ratios (95% CIs) for cancer outcomes associated with 1-SD change in normalized difference vegetation index (NDVI) in 300-m buffers, excluding participants with follow-up shorter than two years.

	N	Cases	Unadjusted	Adjusted	p-for-effect modification
**Cancer incidence**					
Overall	4,104	2,052	0.95 (0.90, 1.01)	0.96 (0.90, 1.02)	.53
No CHD	975	491	1.02 (0.89, 1.16)	0.96 (0.82, 1.12)	
CHD	3,129	1,561	0.94 (0.88, 1.00)	0.96 (0.89, 1.03)	
**Second primary cancer incidence**					
Overall	3,663	272	1.01 (0.91, 1.12)	1.05 (0.94, 1.17)	.56
No CHD	685	58	0.93 (0.73, 1.18)	0.97 (0.73, 1.28)	
CHD	2,978	272	1.03 (0.92, 1.16)	1.04 (0.92, 1.18)	
**Mortality among incident cases**					
Overall	2,052	1,107	0.93 (0.88, 0.99)	0.92 (0.87, 0.98)	.28
No CHD	491	235	0.86 (0.75, 0.98)	0.93 (0.80, 1.08)	
CHD	1,561	872	0.96 (0.90, 1.02)	0.94 (0.88, 1.00)	
**Mortality among prevalent cases**					
Overall	1,611	1,080	0.92 (0.87, 0.98)	0.92 (0.87, 0.98)	.13
No CHD	194	114	0.76 (0.61, 0.95)	0.72 (0.57, 0.91)	
CHD	1,417	966	0.93 (0.88, 1.00)	0.93 (0.88, 1.00)	

* Adjustment for sex, residential socioeconomic status, smoking, chronic exposure to air pollution (NO_x), diabetes, hypertension and prior stroke.

Supplemental Table 6. Hazard ratios (95% CIs) for cancer outcomes associated with 1-SD change in normalized difference vegetation index (NDVI) in 300-m buffers, excluding participants with self-reported coronary heart disease (CHD).

	N	Cases	Unadjusted	Adjusted	p-for-effect modification
**Cancer incidence**					
Overall	4,622	2,311	1.00 (0.94, 1.06)	1.01 (0.94, 1.07)	.76
No CHD	1,102	551	1.03 (0.91, 1.17)	0.97 (0.84, 1.12)	
CHD	3,520	1,760	0.99 (0.92, 1.05)	1.01 (0.94, 1.08)	
**Second primary cancer incidence**					
Overall	3,860	334	1.00 (0.90, 1.11)	1.03 (0.92, 1.16)	.64
No CHD	750	72	0.93 (0.73, 1.18)	0.97 (0.73, 1.28)	
CHD	3,110	262	1.01 (0.90, 1.14)	1.02 (0.90, 1.16)	
**Mortality among incident cases**					
Overall	2,311	1,294	0.94 (0.88, 0.99)	0.92 (0.87, 0.98)	.32
No CHD	551	268	0.87 (0.76, 1.00)	0.95 (0.81, 1.10)	
CHD	1,760	1,026	0.97 (0.91, 1.04)	0.93 (0.87, 1.00)	
**Mortality among prevalent cases**					
Overall	1,549	1,031	0.91 (0.86, 0.97)	0.91 (0.86, 0.97)	.16
No CHD	199	119	0.76 (0.61, 0.95)	0.72 (0.57, 0.91)	
CHD	1,350	912	0.93 (0.87, 0.99)	0.92 (0.86, 0.99)	

[*] Adjustment for sex, residential socioeconomic status, smoking, chronic exposure to air pollution (NO_x), diabetes, hypertension and prior stroke.

Supplemental Figure 1. Heterogeneity analysis for the association between 1-SD change in normalized difference vegetation index (NDVI) and cancer incidence (panel A) and post-cancer mortality among the incident (panel B) and the prevalent (panel C) groups, stratified by organ systems with at least 150 cases. Presented are hazard ratios (95% CI) on log scale, and all models were adjusted for age, sex, residential socioeconomic status, smoking, chronic exposure to air pollution (NOx), diabetes, hypertension and prior stroke.

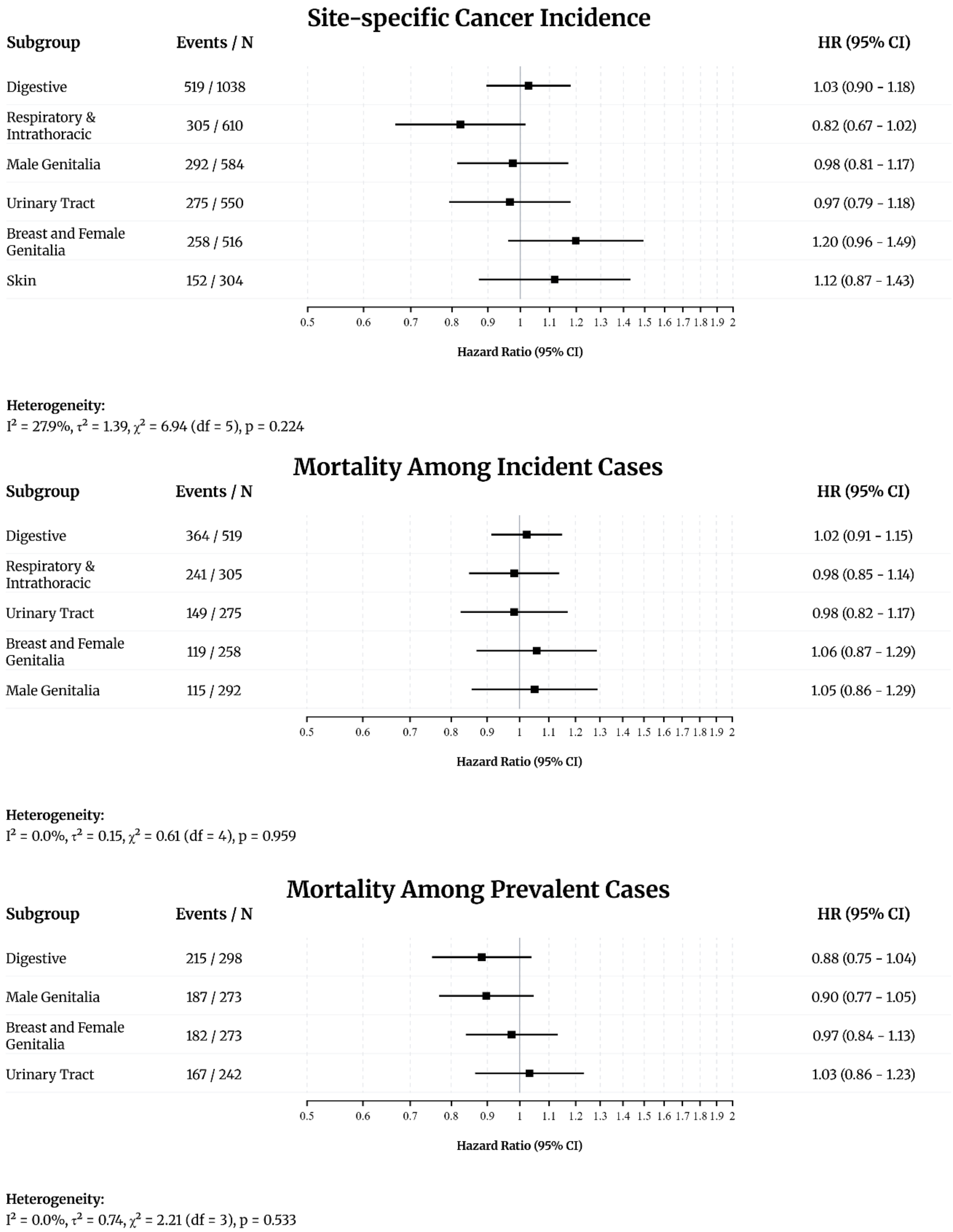

Supplemental Figure 2. Cohort-stratified associations between 1-SD change in NDVI and cancer incidence with 1:1 (Panel A) and 1:2 (Panel B) matching and post-cancer mortality among the prevalent group (Panel C) and the incident group (Panel D). Presented are hazard ratios (95% CI) on log scale; diamonds represent fixed-effects pooled estimates; arrows indicate confidence intervals extending beyond the displayed range. All models were adjusted for age, sex, residential socioeconomic status, smoking, chronic exposure to air pollution (NOx), diabetes, hypertension and prior stroke.

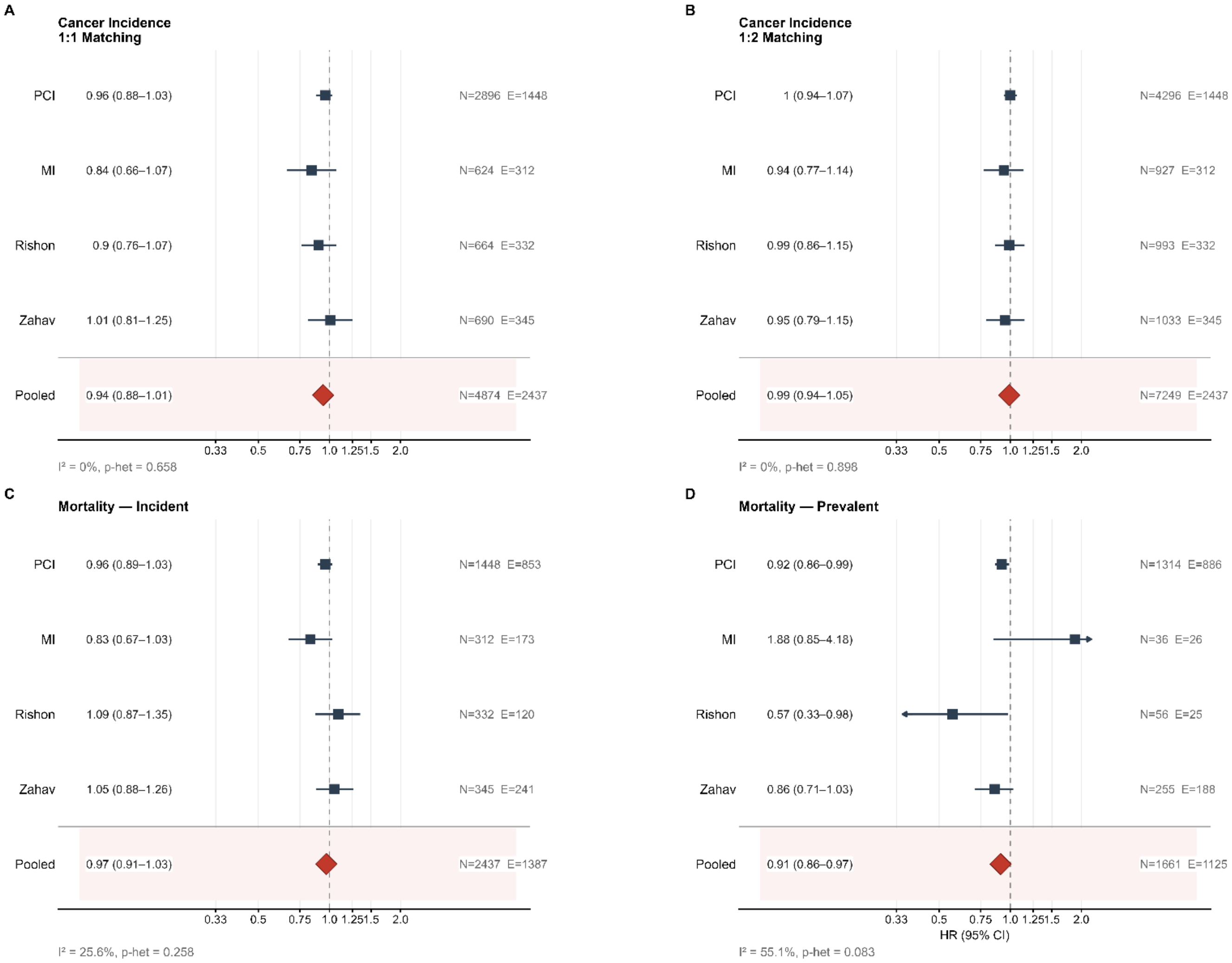

Supplemental Figure 3. Associations between 1-SD change in NDVI and post-cancer mortality by CHD severity stratum within registry cohorts: number of diseased vessels in the PCI cohort among incident (panel A) and prevalent (panel B) cancer cases, and Killip class in the MI cohort among incident cases (panel C). MI prevalnt cases were ommited due to insufficient sample size. Presented are adjusted hazard ratios (95% CI) on log scale. All models were adjusted for age, sex, residential socioeconomic status, smoking, chronic exposure to air pollution (NOx), diabetes, hypertension and prior stroke.

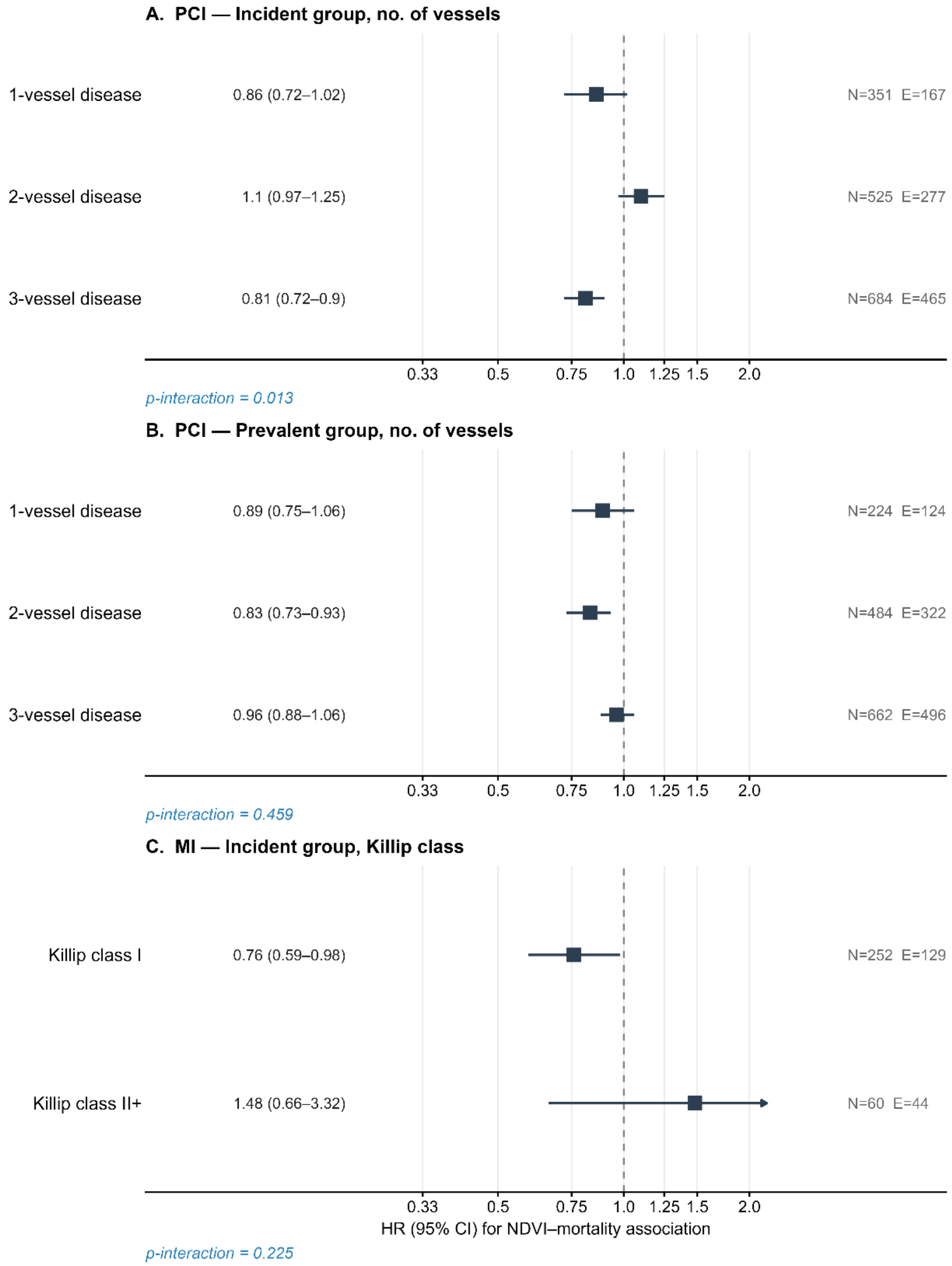

List of R packages used for the analysis

foreign

survival

tableone

tidyverse

dplyr

lubridate

corrplot

survminer

patchwork

DataExplorer

flextable

readxl

DT

glue

forester
